# Supplementary material for: Rehydration Beverages Made from Quercus sideroxyla Infusion, Probiotics, and Prebiotics: Antioxidant and Anti-Inflammatory Potential
Source: Foods. 2025 Feb 28;14(5):837. doi: 10.3390/foods14050837 (PMC11898763; doi:10.3390/foods14050837)
Supplement: Supplementary file 1 [file foods-14-00837-s001.zip › foods-3476012-supplementary.pdf]

Table S1. Storage stability under room temperature conditions of microencapsulated probiotics of the *Akkermansia muciniphila* and *Bifidobacterium longum* strains

Akkermansia muciniphila					
**Time(days)**	**LOG UFC/g**	**aw**	**Humidity (%)**	**Solubility (%)**	**Wettability (min^{-1})**
**0**	10.44±0.02^{a}	0.118±0.001^{a}	3.15±0.07^{a}	94.50±0.45^{a}	4.37±0.04^{a}
**15**	10.43±0.02^{a}	0.122±0.001^{a}	3.2±0.14^{a}	94.11±0.22^{a}	4.24±0.01^{b}
**30**	10.43±0.02^{a}	0.130±0.001^{b}	3.3±0.14^{a}	94.63±0.22^{a}	4.15±0.01^{c}
**45**	10.36±0.02^{b}	0.138±0.002^{bc}	3.4±0.00^{b}	94.37±0.39^{a}	4.13±0.01^{c}
**60**	10.37±0.02^{b}	0.143±0.003^{c}	3.55±0.07^{b}	94.24±0.22^{a}	4.11±0.01^{c}
**75**	10.33±0.02^{b}	0.144±0.004^{c}	3.85±0.07^{c}	94.76±0.00^{a}	4.10±0.00^{c}
**90**	10.33±0.02^{b}	0.159±0.005^{d}	4.1±0.00^{d}	94.37±0.39^{a}	4.10±0.01^{c}
Bifidobacterium longum					
**Time(days)**	**LOG UFC/g**	**aw**	**Humidity (%)**	**Solubility (%)**	**Wettability (min^{-1})**
**0**	10.40±0.04^{a}	0.117±0.002^{a}	2.85±0.07^{a}	96.77±0.46^{a}	4.11±0.01^{a}
**15**	10.39±0.02^{a}	0.121±0.000a	3.15±0.07b	96.11±0.23^{a}	4.09±0.00a
**30**	10.37±0.02^{a}	0.147±0.002b	3.45±0.07c	96.50±0.23^{a}	4.09±0.01^{a}
**45**	10.36±0.02^{a}	0.178±0.001c	3.55±0.07c	96.37±0.23^{a}	3.59±0.01b

**60**	10.33±0.02b	0.181±0.001d	3.80±0.00d	96.11±0.23ª	3.55±0.01c
**75**	10.29±0.03b	0.204±0.003e	4.15±0.07e	96.63±0.00a	3.55±0.00c
**90**	10.30±0.00b	0.221±0.002f	4.45±0.07f	96.11±0.46ª	3.54±0.00c

Diferencias significativas expresadas por literales distintas de acuerdo con Tukey (α=0.05).
